# The Systematic Review of Artificial Intelligence Applications in Breast Cancer Diagnosis

**DOI:** 10.3390/diagnostics13010045

**Published:** 2022-12-23

**Authors:** Dilber Uzun Ozsahin, Declan Ikechukwu Emegano, Berna Uzun, Ilker Ozsahin

**Affiliations:** 1 Medical Diagnostic Imaging Department, College of Health Sciences, University of Sharjah, Sharjah 27272, United Arab Emirates; 2 Operational Research Center in Healthcare, Near East University, TRNC Mersin 10, 99138 Nicosia, Turkey; 3 Department of Statistics, Carlos III University of Madrid, 28903 Madrid, Spain; 4 Department of Mathematics, Near East University, TRNC Mersin 10, 99138 Nicosia, Turkey; 5 Brain Health Imaging Institute, Department of Radiology, Weill Cornell Medicine, New York, NY 10065, USA

**Keywords:** artificial intelligence, breast cancer, diagnosis

## Abstract

Several studies have demonstrated the value of artificial intelligence (AI) applications in breast cancer diagnosis. The systematic review of AI applications in breast cancer diagnosis includes several studies that compare breast cancer diagnosis and AI. However, they lack systematization, and each study appears to be conducted uniquely. The purpose and contributions of this study are to offer elaborative knowledge on the applications of AI in the diagnosis of breast cancer through citation analysis in order to categorize the main area of specialization that attracts the attention of the academic community, as well as thematic issue analysis to identify the species being researched in each category. In this study, a total number of 17,900 studies addressing breast cancer and AI published between 2012 and 2022 were obtained from these databases: IEEE, Embase: Excerpta Medica Database Guide-Ovid, PubMed, Springer, Web of Science, and Google Scholar. We applied inclusion and exclusion criteria to the search; 36 studies were identified. The vast majority of AI applications used classification models for the prediction of breast cancer. Howbeit, accuracy (99%) has the highest number of performance metrics, followed by specificity (98%) and area under the curve (0.95). Additionally, the Convolutional Neural Network (CNN) was the best model of choice in several studies. This study shows that the quantity and caliber of studies that use AI applications in breast cancer diagnosis will continue to rise annually. As a result, AI-based applications are viewed as a supplement to doctors’ clinical reasoning, with the ultimate goal of providing quality healthcare that is both affordable and accessible to everyone worldwide.

## 1. Introduction

Breast cancer (BC) is one of the greatest threats to women in the 21st century. It has rendered many women mentally unstable [1] and many lives have been lost. Although early treatment reduces the mortality rate of this malignancy, a late diagnosis is potentially fatal. Breast cancer’s pathogenic effects include age [2], reproductive effects such as breastfeeding [3], testosterone levels and menopause [4], familial history, genetic disorders [5], and other environmental effects [6]. Anatomically, the breast is made up of healthy blood vessels, connective tissues, milk duct lobules, and lymph nodes. However, breast cancer occurs when abnormal cells (tumors) grow in the connective tissues, milk ducts, lymph nodes, and lobules of the breast [7]. Breast cancer can be benign or cancerous. Cancerous can be classified by invasive carcinoma and non-invasive carcinoma [8]. Invasive carcinoma is highly pathological with metastatic [9] adverse conditions while non-invasive breast cancer does not cause proliferation to the neighboring organs [10]. Meanwhile, according to a 2020 statistical evaluation [11], approximately 2.3 million women were diagnosed with breast cancer, resulting in 685,000 deaths, globally [12]. In the United States of America, approximately 13% of women will experience breast cancer in their lifetime. Annually, 287,850 invasive cases and 51,400 non-invasive cancers were recorded [11]. The incidence has increased tremendously, however, the mortality rate has recently been reduced [13]. Concurrently, the incorporation of artificial intelligence (AI) into the diagnosis of breast cancer is an emerging procedure that results in a better diagnosis of the disease. AI’s success rate has been attributed to its ability to reproduce, using high-resolution images from variable tissue specimens. AI is the application of computational models to simulate human intellectual abilities as well as resolving severe healthcare concerns, such as complicated biological anomalies such as cancers [14]. Numerous genomic and epigenomics variants contribute to cancer’s complexity and diversity; as a result, the use of AI algorithms to diagnose gene mutations or these abnormalities in protein complexes at a preliminary phase has enormous promise. This study’s objective is to investigate the recent developments in diagnostic applications of AI in breast cancer diagnosis by assiduously surveying the current literary studies according to preferred reporting items for systematic review and meta-analysis of diagnostic test accuracy (PRISMA-DTA).

### AI in Breast Cancer

At present, AI has been introduced into practically every industry to maximize production, efficiency, and accuracy [15,16]. Advances in computation, data, and algorithm performance have made AI more powerful, user-friendly, and objectively directed than previously. It is also utilized for intrusion detection systems [16], pictorial synthesis [17], optical character recognition (OCR) [18], facial expression identification, etc. In healthcare, AI is applied in different domains, such as patient monitoring [19], drug dispensing, and hospital management [19,20]. AI has undeniably had a greater impact on complex image analysis, as well as providing data for quantitative assessment through automation and removing the radiation risk associated with breast radiological examination [21]. To mimic the rational decisions of humans with operational excellence, AI offers superintelligence; using AI techniques can be beneficial for the instant incorporation of feature learning, the ability to process and handle complex and multiple dimensional data, as well as its availability of diagnostic data from a variety of clinical experiments. Therefore, medical consultants, academicians, and oncologists have recognized the potential of developing and employing AI in many parts of the diagnosis of breast cancer disorders. This hope has been fueled by recent advancements in AI [21]. AI research often uses enhanced models with higher cancer incidence than screening programs. As a result, algorithms for imaging assessment and the diagnosis of breast cancer, in each category of AI algorithms, have demonstrated substantial improvements. Meanwhile, deep learning (DL) algorithms have also been discovered to be substantially more encouraging than conventional machine learning (ML) algorithms [22,23]. They have also demonstrated that they are strong candidates for pre-existing chronic imaging studies, and particularly for breast cancer image processing research [22,23]. Alternatively, Buckner et al. summarized ML, computer-assisted detection, and computer-assisted diagnosis as now being possible. It reduces the number of false positives (FP) that are produced by the outputs from the Chan-Vese segmentation technique after it has been initialized using the marker controller watershed algorithm (MCWS). The computer-assisted diagnosis technique makes use of blended learning, which consists of four Support Vector Machine (SVM)-based classifiers. Each of these base classifiers uses the features extracted from a certain tissue constituent [24]. There are possibilities, on the other hand, that mammography misses many malignancies because of several factors, such as breast density, tumor size, or subtle indications of cancer that are invisible in cases of metastasis. AI is accurate in detection using digital mammography. In addition, the sensitivity of AI, when evaluated in terms of its ability to pick up on the various breast cancer morphological characteristics, such as, asymmetry, and distortion, proves highly effective [25].

The AI algorithm is used in medical imaging. Although picture archiving and communication systems (PACSs) are now in use, and constitute a tremendous supply of information, the practical use of AI in medical imaging has been hampered by the absence of large public databases. Despite this, a significant number of software programs that are extremely helpful for diagnoses in general, and particularly for the identification of breast lesions, have been established. The AI detection of lesions is automatically applied in a variety of imaging techniques, and it is now the most prevalent AI application. It involves locating the regions of the image that have high and different lesions, based on the training of the models. Recent research has shown that convolutional neural networks (CNNs) are capable of matching the detection capability of an experienced radiologist [26]. CNN categorization has the benefit of removing any variabilities. The delineation of the boundaries of the lesions is a vital reason for the application CNN in lesions [27]. CNN, when simulated to complex tasks, performs with higher accuracy and speed than humans [28]. As a result, U-nets, which are utilized for the segmentation of the images, is a typical example of the kind of network that is employed for this objective; they can differentiate between tissues, such as glandular and adipose, in digital mammography after the volume of the lesion is calculated [29]. On the other hand, the diagnostic criteria for classifications in radiography and histopathology are widespread; nonetheless, the manual identification, classification, and grading employed by radiologists are time-consuming and more prone to inter and intra-observer variances. However, improved clinical outcomes rely solely on the early identification of breast cancer [30]. According to the National Breast Cancer Foundation, this early diagnosis involves breast lumps, mass, density, etc., being detected at an early stage, when it is still in the localized stage. If early detection is achieved the overall survival rate after five years is 99%. Therefore, performing monthly breast self-examinations, in addition to performing periodic clinical breast examinations such as mammography, is an important part of early detection [31] and it increases a patient’s chance of surviving the disease [32]. The study by Jan Witowski, Laura Heacock, et al. [33] created a DL methodology that increases the specificity of the dynamic contrast-enhanced magnetic resonance imaging (DCE-MRI) of breast tissue. DCE-MRI is a technology that is occasionally utilized for women who are at a higher risk of developing breast cancer. These scientists verified the DL techniques on different cohorts, which demonstrated that this strategy has the potential to reduce the number of needless surgeries by minimizing the percentage of false positives [33]. The identification of breast cancer at an earlier stage is essential for improving treatment outcomes. On the other hand, AI is a tool that improves breast cancer screening. The application of screening strategies using AI on a population has proved effective in lowering the mortality rate caused by breast cancer. The use of AI technology in screening cancer achieves 89% specificity and 76% sensitivity, both of which are substantially higher compared with the figures for standard computer-aided diagnosis (CAD) systems, which are approximately 50% [34]. This was achieved using digital mammography, which serves as the screening method for the women invited to take part in the screening programs. The use of digital mammography allows for the ability to construct CAD systems [35] that have the potential to lessen the workload of radiologists. Studies have indicated that AI-driven technologies have superior diagnostic accuracy than conventional techniques, and this trend toward the widespread implementation of AI-based systems will likely continue [35]. When it comes to helping radiologists in the interpretation of digital mammograms in the Breast Cancer Early Detection Program (BCEDP), recent advancements in AI have opened up possibilities that extend farther than what is provided by standard CAD systems. The goals of AI systems are similar to CAD systems: enhancing the diagnosis of malignant tumors, minimizing the effects of interval malignancies, and, simultaneously, minimizing the amount of reading required. It is possible that, in the long run, new AI systems and methods will even increase the expense ratio of BCEDPs [36].

In another literary study, the distinction between benign and malignant tumors was made through the use of ultrasonography, which is a form of digital imaging. The identification of breast cancer by breast ultrasonography has been suggested to benefit from the application of several different AI techniques. At present, great classification performance in biomedical images can be achieved with the application of various learning approaches, particularly DL. The image classification model offers an accuracy of 97.18% and could classify breast cancer as normal, benign, or malignant [37]. In the same manner, traditionally, ultrasound has been the diagnostic method of choice for determining whether breast tumors are benign or cancerous. As it detects occult breast malignancies, it has become an emerging procedure in this modern time. In contrast to other techniques, such as mammography, DBT, and MRI, ultrasound has some advantages, for example, it is non-ionizing, affordable and has the capacity to provide detailed insights and surveillance [38]. According to the National Breast Cancer Foundation’s 2020 report, AI has been used successfully in the diagnosis of more than 276,000 breast cancer cases. In addition to this figure, 48,000 cases were diagnosed using the non-deep learning methodology, particularly in the diagnosis of aggressive cancerous types. Gene testing and histopathological imaging are two methods that can be used to identify breast cancer. As genetic analysis is not cost-effective, medical laboratory histological imaging is most frequently employed for breast cancer screening and diagnosis. The combination of action DL and ML enables the comprehensive analysis of the diagnosis and treatment of breast cancer using genetic sequencing or histopathological imaging. Breast cancer image analysis using AI can detect breast lumps (masses), mass segmentation, breast density, and the risk of breast cancer. In the majority of patients, breast lumps are the most frequent occurrence of breast cancer [39]; consequently, their detection is an essential step utilized in CAD [40]

The studies by Farahnaz Sadoughi et al., in which breast cancer was diagnosed using image acquisition, was the first step to be taken in the image process, then the image is processed and segmented. Different AI techniques, such as SVM, KNN, genetic and Naive Bayes, as well as DL [41,42,43], were employed for its categorization. According to the findings, SVM had the highest accuracy percentage across the board for all of the different image analysis tasks. In order to enhance the diagnostic efficacy, Shahid Munir Shah, et al. utilized a variety of imaging techniques to streamline the process of breast cancer identification. These imaging techniques include mammograms, ultrasound (US), magnetic resonance imaging (MRI), histopathological pictures, or any hybrid model of the aforementioned imaging techniques. The development of powerful AI algorithms, such as DL, and the accessibility of large datasets are two factors that have contributed to the recent uptick in scientific research [44]. In addition, Dileep G. [45] and S.M. Shah [46] used several imaging modalities in the diagnosis of breast cancer, such as mammography, X-ray, thermography, magnetic resonance imaging (MRI), Positron computed tomography (PET), computed tomography (CT), ultra-high-density ultrasound and histological examinations. The results from using these imaging modalities showed that most women have malignancy, whose etiologic factors are linked to heredity, lifestyle, and environmental. AI is employed in breast cancer diagnosis because it yields faster, more accurate diagnoses.

Sha et al. [46] summarized breast cancer in terms of etiology, diagnosis, and treatment, as well as preventive measures. These are called prediction classifiers and models that categorize a woman’s chances of acquiring breast cancer, as well as direct screening guidelines supporting the existence of established and quantified hormonal, ecological, behavioral, or familial risk variables. This familial factor contributes to the development of breast cancer in women. However, image modalities quantification such as magnetic resonance imaging (MRI) is a proven effective diagnostic model, particularly in the diagnosis of this malignancy. Mammography is the primary diagnostic choice [47] that many health personnel utilizes as it creates a significant level of specialization for instance molecular and genomic profiling that are essential in the management of breast cancer [46]. Furthermore, additional studies have shown the relationship between the image analysis of breast cancer and its application; the diagnosis of BC, segmentation techniques, camera calibration, and data processing are all parts of this process. Studies have also shown the successes and application span of supervised and unsupervised learning such as DL, CNN, and other related approaches in breast cancer evaluation. The combination of unsupervised and transfer learning (TL) in BC diagnosis is an emerging technique in AI. TL has the potential to ease, albeit only slightly, the problem of insufficient annotations of data. Utilizing a CNN that has already been trained on the approaches an organization takes throughout the procedure of TL is more effective than carrying out further supervisory training [48]. It is necessary to utilize numerous modes or types of images in the registration and fusion when performing diagnostic and therapeutic analysis of breast cancer. This helps clinicians to gain more knowledge, which helps them in diagnostic accuracy [49]. The registration of images in an AI breast cancer diagnosis locates reference points in a plurality of images; this is accomplished by performing rotation (spatial) on the images to place them to t in a coordinate. This must coincide exactly, one for one, for registration to be successful [50]. The utilization of computational methods including digital technologies (image analysis and AI), work in conjunction with X-rays to assist in the early detection of BC. The expansion of this, coupled with the development of high tech, has not only facilitated the disease’s earlier diagnosis but has also made it possible to treat a significant number of patients [51].

The diagnostic significance of images is improved by the process of fusion, which involves the extraction of meaningful information from many images, and the filtering of unnecessary information, and consequently the improvement of image quality. In general, signal level, data level, feature level, and decision level fusions make up the process of the fusion of images, in ascending order, from lowest to highest [52]. The use of meta-analysis in the diagnostic efficacy of DL has enabled the timely identification of breast cancer. The study used four categories: breast cancer; validation type; imaging modalities, such as, ultrasound; and DL algorithms versus healthcare professionals. The results showed that the pooled sensitivity was 88% (95% confidence interval: 85–90%), the specificity was 84% (79–87%), and the AUC was 0.92. (0.90–0.94). It was shown that all of the subgroups had a diagnostic accuracy that was satisfactory when using equivalent DL algorithms. Consequently, these techniques are beneficial for detecting breast cancer through the use of diagnostic imaging [52]. Again, Xue et al. [53], Freeman et al. [54], Mendes et al. [55] in all the three studies, AI was shown to detect 53, 45, and 50 percent of low-risk cancer, respectively. Additionally, AI identified 10%, 4%, and 0% of the breast cancer data from the data set that has already been screened and is utilized by radiologists. In the application of AI to BC images, AI presented a summary of the advantages and problems during the BC imaging survey, including prospective solutions, utilizing ML [56] and DL to forecast the risk of cancer, mammography appraisal, and data set labeling. In addition, the systematic and rapid segmentation of the region of interest (ROI) and breast density in magnetic resonance imaging (MRI), as reported by Pandey et al. [57], showed that the pictorial-based analysis displayed excellent segmentation in terms of accuracy, specificity, AUC, and sensitivity. The success was made evident by many technologically advanced devices which use the principle of DL for the diagnosis of BC. In particular, (AI)-CAD systems, such as iCAD’s PowerLook Tomo Detection and Screen Point Medical’s Transpara, are emerging into existence as the utilization of computed tomography becomes more widespread [57]. In summary, according to Shah et al. [44], many Al applications in BC diagnosis were reported by Nassif et al. [58], Dileep and Gianchandani Gyani [45], Huang et al. [32]., H-p et al. [59] and Shah et al. [59], whose evaluations offers a promising remark that the challenges of cancer prognosis and diagnosis are dealt with using advances in AI. These challenges are resolved through the use of AI, as AI has shown higher diagnostic accuracy than CAD in the detection of BC in mammograms. In addition, because the data set is readily available, AI may now be utilized to analyze mammograms using various image processing techniques. As a result of their lower cost and increasing prevalence, the images obtained from histological breast cancer tests are utilized in DL BC detection. AI has been integrated into several screening procedures to determine breast mass, density, and segmentation. The overall summative description of the various literary studies is given in Table 1.

## 2. Methodology

**Figure 1:**
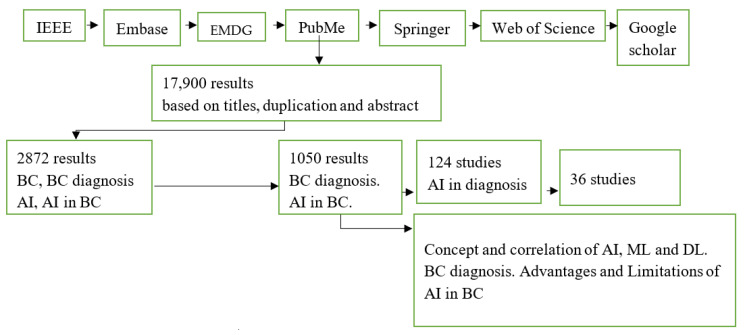
Block diagram of AI in BC diagnosis.

The methodology employed in this systematic review is devoid of any medical (either prospective or retrospective) data of patients; therefore, it was not necessary to obtain ethical approval to carry out the study. The data used in this study are articles from open-access publications based on details such as the dates of publication, authors’ names and methodologies used, type of AI models, dataset employed, and the overall results, such as the area under the curve (AUC), sensitivity, specificity, and accuracy. The literature searches were based on journals published in English between 2012 and 2022, all-inclusive. Breast cancer, breast cancer diagnosis, AI, and AI in breast cancer are the keywords used in the search. In the review, we searched these databases: IEEE, Embase, Excerpta Medica Database Guide Ovid, PubMed, Springer, Web of Science, and Google Scholar. A total of 17,900 results were found based on the aforementioned keywords. Some of the search results were out of the scope of breast cancer and/or AI. Those that were with the scope of this study had treatment options instead of diagnosis which led to further screening. Ultimately, 36 studies relevant to this review were used in this study. A detailed summary of the findings is shown in Table 1. A subcategory with a detailed explanation of BC diagnosis advantages, disadvantages/limitations, and AI in BC are detailed, as can be seen from the block diagram (Figure 1) of this review.

## 3. Results and Discussion

**Figure 2:**
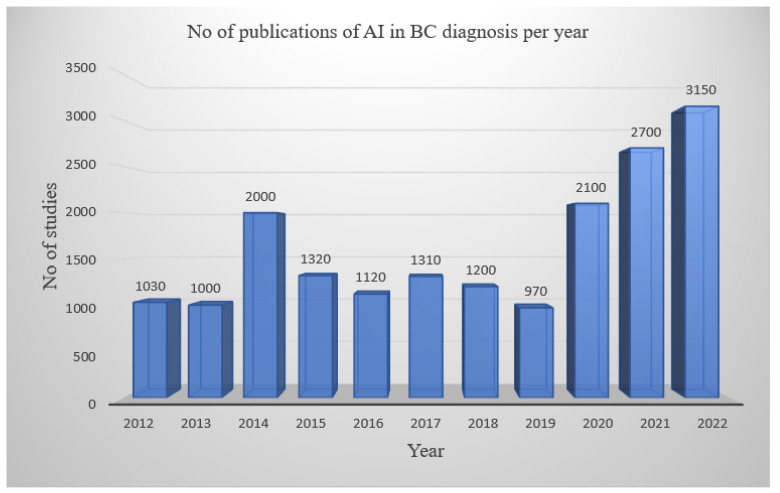
Showing publications on AI in breast cancer diagnosis between 2012–2022.

This study demonstrated the various articles that were systematically reviewed concerning the application of AI in breast cancer diagnosis, as published in various journals. Recently, there has been an increase in AI-based studies on the diagnosis of BC, which has demonstrated a significant value in this study. Rowland. W. [39] had a similar study on the concept and correlation of AI, ML, and DL, however, the application was geared towards other clinical predictions instead of BC specifically. Figure 1 shows the block diagram for this review. The selected studies were published between 2012 and 2022 and are all-inclusive. Figure 2 shows the annually published data on AI in BC diagnostics from the various aforementioned databases. As a result of AI’s promising use and modifications to suit breast tomosynthesis, there have been many studies on the applications of AI in BC diagnosis, from 2020 to date [40]. Meanwhile, the vast majority of the studies in Table 2 show prediction classification models in the evaluation of the parameters.

**Figure 3:**
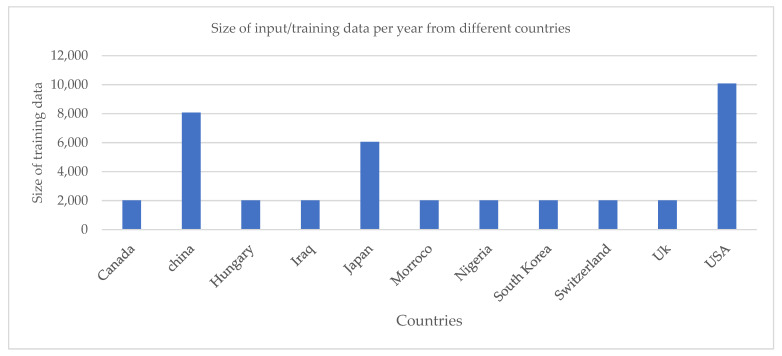
Size of input or training data used by the different countries where the studies were carried out.

**Figure 4:**
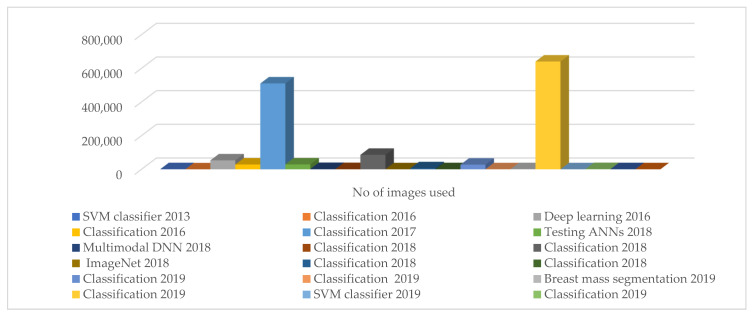
Summary of task, date, and number of images or studies used in figure.

In addition, Table 3 shows the comparison of the latest related studies. However, accuracy (99%) has the highest number of performance metrics, followed by specificity (98%) and the area under the curve (0.95), as reported by Berker et al. [41]. These authors [42,43,44], use the CNN and the Digital Database for Screening Mammography (DDSM) in the comparative analysis of ImageNet and the classification of breast cancer [45,46]. Similarly, the author uses a Deep Neural Network (DNN) to classify breast cancer. As is evident from this review, the majority of the studies have focused on specificity or area under the curve without other parameters such as sensitivity, the accuracy of the diagnostic procedure, etc. being mentioned. This is a typical example of the limitations in medical studies. However, models such as CNN, Deep Convolutional Neural Networks (DCNN), Artificial Neural Networks (ANN), Digital Databases for Screening Mammography (DDSM), and others have been used in various studies. This is because these models are capable of automatic cancer detection as well as lesion interpretation. The studies provide independent advice for radiologists and oncologists to improve lesion identification and prognosis. Meanwhile, Figure 3 shows that the USA has the highest number of studies, followed by China and Japan. However, Figure 4 showed the summary of the task, date, and number of images or studies in the summary of Table 2. i.e., various algorithms of AI applications and their performances in breast cancer diagnosis. The figure shows that the classification algorithm was mostly used in the years between 2016 and 2019. In addition, in 2017 and 2019, 510,000 cases of fibro glandular breast density and 640,000 cases of breast imaging were studied using a classification algorithm in Switzerland and the United Kingdom, respectively.

### 3.1. Emerging Techniques of AI Applications in BC Diagnosis

Radiomics is an emerging technique that extracts variable quantitative features from images of medical origin [86]. Radiomics has been useful in the management and diagnosis of BC. The knowledge of radiomics helps in predictions, the staging of tumors, and the evaluation of therapeutic response [87]. Many academicians [88] have agreed that radiotherapy is very effective in the prognosis of BC. However, the only shortcoming of radiomics is that the data generated from locally advanced breast cancer (LABC) after non-adjuvant therapy and during post-surgical therapy cannot predict the survival rate of BC. Meanwhile, using AI’s application in BC diagnostics, reproducibility serves as a key point of experimental science, yet could be hampered by many factors such as the human or biological system, intrinsic variables, mislabeled samples, cross-contamination, and in some cases, over-passage in the cellular lines [89]. Aside from the generalizability issue, researchers use methods to remove these unnecessary (irrelevant) factors, which heavily rely on statistically applicable facts [88]. Generalizability issues are commendable trends, and the data set generated could be processed before mining [90,91,92]. This has demonstrated the importance of preprocessing medical data sets. However, this study has numerous unutilized social benefits. According to [93], AI applications in BC are becoming more common in developing countries. The reduced cost of the DeepMind automated system, for instance, offers relief to both developed and underdeveloped countries, as recognized by the World Health Organization (WHO) [93].

### 3.2. The Conception and Respective Correlations of AI, ML, and DL

**Figure 5:**
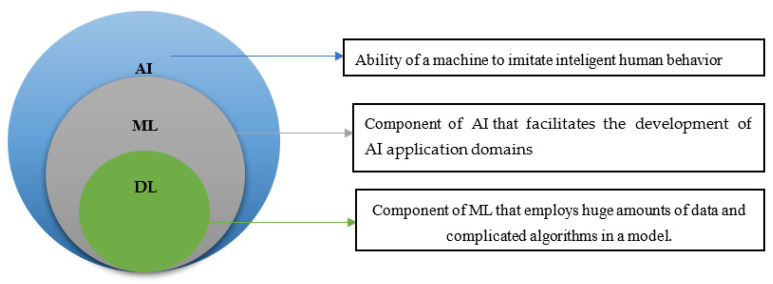
Relationship between AI, ML, and DL.

AI includes techniques that enable computers to mimic the behavior of humans. The primary functions of AI in breast cancer screening are the segmentation and classification of benign or cancerous tumors [94]. ML is a learning algorithm whose characteristics and variables represent observable data [95]. DL is a typical example of ML, the methodology of which is dependent on deep neural networks that resemble but exaggerate human brain neurons [95] and are used in the classification and recognition of images [95]. DL uses a deep modular structure to promote hierarchies in learning and extracts information from simple to sophisticated models. However, there are several distinctions between ML and DL. Also, Figure 5 shows the relationship between AI, ML and DL. In terms of data dependencies, ML learns a mathematical model from training data [96]. The learned model forecasts the future by testing the data. Learning or training in ML denotes the collaborative technique of assessing the discrepancy between malignancy and benign using an assessment metric called the objective function [97]. This learning could be supervised learning [97], whereby the observed training data and the target are prerequisites for a training model. As a result, several research laboratories and corporations are attempting to build AI technologies for diverse healthcare domains. Supervised Machine Learning (SML) [98] may give healthcare professionals improved assistance in conducting differential diagnosis. The SML methodology also employs sophisticated ways to forecast health problems and alert the entire public towards impending danger [99]. In mammography, the cancer images are labels that allow the algorithm to learn the features of this malignancy. However, unsupervised learning [100] has no diagnostic features or abnormal labels. In semi-supervised learning, the information provided for the algorithm is not important for the training [101]. Simultaneously, the additional methodology could be employed to improve efficiency and cut down on the number of inaccurate predictions about breast cancer. The same can be said for breast pictures used to potentially detect breast cancer [35,102,103,104]. When compared to manual methods, AI-based automated image analysis helps eliminate laborious and time-consuming screening processes, while also efficiently capturing useful and relevant information from large amounts of image/picture data. This is accomplished in comparison to manual inspection.

### 3.3. BC Diagnosis Advantages

When AI is used in mammography, it can detect cancer up to two years earlier than a human oncologist. If diseases can be identified and treated sooner, more lives can be spared. Doctors can be more certain in their diagnoses with the aid of AI, thus increasing their efficiency [55]. AI also reduces the stress encountered by radiologists. Instead of spending hours reading mammogram images, AI is never tired. With AI, accuracy and earlier cancer detection are achieved. In addition, with the help of AI, medical care can be enhanced [56]. The use of AI-based diagnostic tools in the diagnosis of breast cancer has helped increase the efficiency of radiologists and produces results that are better than those obtained by radiologists working alone. While AI distinguishes between structures and image components using complex ML algorithms, its application in clinical practice is limited due to its low specificity [57]. In particular, with the use of AI, the accuracy of CAD has increased. AI uses CNN, a DL technology well-suited for image assessment and classification that has diagnostic accuracy that is approximate to or even better than that of radiologists [59] in cancer detection. AI could also help diagnose breast cancers that are not identified in the screening; as a result, there is a need for further screening, especially in dense breast tissues, which have low sensitivity. Some studies have shown that dense breasts have a higher rate of false-negative outcomes [58]. A negative mammography result may reassure women when they have cancer. The use of AI in the diagnosis of BC is saddled with problems. This makes AI limited, as can be seen in the subsequent section.

### 3.4. Limitation of AI in BC Diagnosis

This is the boundary between AI and human intellect. There is a need for AI to independently produce, replicate and accept data without human aid. AI machines could be considered a legitimate form of AI. This necessitates the existence of a universally accessible code, which can only be accomplished through the equitable distribution of data [105]. Databases that are simple to navigate and software that is intuitive to use need to be implemented into the information technology systems of hospitals across the globe. Additionally, there should be trust and confidence among clinicians to henceforth implement AI in all of their clinical decisions. Consequently, medical professionals should have adequate training on the use of AI innovations. The modern era and technology have provided us with apps that monitor diseases, such as heart rate and diabetes; however, this is yet to be seen in cases of BC. This will improve the quality of patient care and patient satisfaction if innovated. Ethically, the confidentiality of data, privacy violations, patient autonomy, consent, etc., are concerns that should be considered when utilizing AI in breast cancer diagnosis. Several precautionary measures are developed to prevent the disclosure of personal information, and legislation is in place to prohibit any misconduct. In addition, radiomics is still lacking widely in clinical practice today [106]. We, therefore, hope that decision-makers will implement these limitations so that AI will remain an effective diagnostic tool.

## 4. Conclusions

This study followed the standard method for the systematic review of papers. Stringent measures were considered in the inclusion criteria. In these criteria, geographically, the test scores were validated, i.e., the articles’ different centers in different countries. This excluded a large volume of studies, particularly in cases where the dataset was used for similar testing and validation. Internal validation overestimates the accuracy and has limited generalizability. This can also result in overfitting, and the loss of generalizability because the model’s performance is dependent on the data. Temporal validation offers a great capacity for the statistical models to forecast future circumstances for the entire population, based on which the model originally derived, after the observations that were used to generate the model. Nevertheless, the non-English studies were excluded because they have no relevance to the study. Meanwhile, the overall diagnostic performance can be improved through the use of AI in diagnostic approaches, for example, in detecting metastatic breast carcinoma in lymph node biopsies. Several models, such as CNN, and Digital Database for Screening Mammography (DDSM), were applied in order to achieve a timely and accurate result. In addition, AI achieves a significant result in images of breast cancer. This study showed that CNN was the most widely used algorithm. In addition, accuracy (98%) has the highest number of performance metrics, followed by specificity (99%) and area under the curve (0.95), and the findings from this study also showed that the majority of the studies were from the United States of America, followed by China and Japan.

## Figures and Tables

**Table 1:** Summary of the results of 36 studies.

Author’s Name	Year	Title	Summary of Result
Kooi et al. [52]	2017	The advantages of Large-scale DL for CAD of breast lesions	The result showed that CNN performs better than CAD in a low sensitivity when compared.
Pandey et al. [57]	2018	The systematic and rapid segmentation of the region of interest (ROI) and breast density in MRI	The pictorial-based analysis displayed excellent segmentation in terms of accuracy, specificity, AUC, and sensitivity
Sadoughi et al. [60]	2018	AI methodology in the diagnosis of BC	The result showed that SVM has the highest accuracy (100%) when compared with various images from thermographs (99.85%), mammograms (93.69%), and ultrasound (US) (95.85%)
Le et al. [61]	2019	The use of AI in breast imaging	The transition from the traditional rule-based CAD to an enhanced embedded knowledge that reduces diagnostic error and improves radiologist accuracy.
Huang et al. [32]	2020	AI in BC, prognosis, and diagnosis	The challenges of cancer prognosis and diagnosis are dealt with using advances in AI
H-p et al. [59]	2020	Combination of AI and CAD in BC. Recent advancements and difficulties	DL employed in clinical image datasets combined with CAD provides a novelty workflow in clinical practice
Shah et al. [44]	2021	AI in detecting BC	AI showed higher diagnostic accuracy than CAD in the detection of BC in mammogram
Freeman et al. [54]	2021	Utilization of AI for image analysis in BC screening initiatives: a comprehensive assessment of the diagnostic validity	In the 3 studies, AI detects 53%, 45%, and 50% of low-risk cancer. In addition, AI detects 10%, 4%, and 0% BC data from the already screened data set used by radiologists.
			Utilization of AI for image analysis in BC screening initiatives: a comprehensive assessment of the diagnostic validity
Shah et al. [44]	2022	Trend and direction in the application of AI in the diagnosis of BC	The availability of the data set makes it possible for AI to be applied using image modalities to the mammogram.
Nassif et al. [58]	2022	The use of AI BC detection	Images from histopathological BC examinations are less expensive and very common, therefor they are used in DL BC detection
Dileep and Gianchandani Gyani [45]	2022	AI application in BC screening and diagnosis	Incorporation of AI into different screening methodologies to detect breast mass, density, and segmentation.
Shao et al. [62]	2022	The application of AI to the clinical study of BC	The AI was able to classify BC according to the many kinds of data, such as plain radiographs, cancer genes, health records, pharmacological information, and biological works of literature.
Mendes et al. [55]	2022	An Overview of the Role of AI in Imaging analysis of BC and Its Various Applications	AI application in BC images using ML, DL to predict the risk of cancer, mammogram evaluation, and labeling of data set, AI showed an overview of the advantages and challenges including the potential solution during the BC imaging survey

**Table 2:** Various algorithms of AI applications and performances in breast cancer diagnosis.

Reference	Date	Task	Country	Tumor Type	Model Source	No Images/Studies	Type of Model	Size of Input/Training	Independent Test Set	Performance Validation	The Area under the Curve (AUC)	Sensitivity (%)	Specificity (%)	Accuracy (%)
Tan et al. [63]	2013	SVM classifier	USA	Breast mammogram	Gaussian kernel	994 women	Gaussian kernel	349 benign, 362 cancerous	NA	NA	0.73	NA	NA	NA
Qiu et al. [64]	2016	Classification	USA	breast	CNN	270	CNN, CAD	200 cases, 70 cases	NA	NA	0.70	70	60	71.4
Ayelet Akselrod-Ballin [65]	2016	DL	Canada	Histopathologica + mammography	R-CNN	52,936	In house Lab	9611	NA	1055	0.72	87.0	77.3	91.0
Samala et al. [66]	2016	mass detection and classification	USA	Mammography + X-ray	SFM and DM	28,330	Cuda- convNet	28,330	94	NA	0.81	83.0	91.0	NA
Becker A, [67]	2017	Classification	Switzerland	Fibro glandular volumetric breast density.	Quantra 2.2	510,000	ANN + Volpara Density	Group 1 = 95/95, Group 2 = 83/513	NA	NA	0.79	78.0	84.0	81.0
Kim E.et al. [68]	2018	Testing ANNs	South Korea	Breast	DIB-MG mammography	29,107	ANN	18 cancers and 233 controls	NA	NA	0.99	73.7 versus 66.6	72.0 versus 92.7	NA
Sun et al. [69]	2018	Multimodal DNN	China	Breast cancer	Metabric	1980	DNN	1054	NA	NA	8.40	95	99	2.4
Mohammed et al. [70]	2018	classification	Iraq	Ultrasound Breast Images	OI + ANN classifier	1393	ANN classifier	900. 300	NA	NA	NA	79.4	84.76	82.0
Ribli D. et al. [71]	2018	classification	Hungary	Mammograms	CNN	86,000	CNN	7700, 847	NA	NA	0.85	98.7	99.6	98.7
Jiao et al. [72]	2018	Alex Net + ImageNet	China	DL Classification	DDSM 300 images	300	DDSM + MIAS set	300	150	150	NA	NA	NA	DDSM = 97.4 MIAS = 96.7
Chougrad et al. [73]	2018	Comparative image classification	Morroco	Mammography + X-ray	DDSM, INbreast, BCDR	6116	DDSM, INbreast, BCDR	641, 688DDSM 300.300INbreast 344,300BCDR	113	NA	0.99	NA	NA	98.2
Wang H et al. [74]	2018	classification	China	Mammography	MV DCNN	736	MV-DNN, + MAP	368	295	74	DNN = 0.828, MAP = 0.846	NA	NA	NA
Lehman et al. [75]	2019	Breast segment classification	USA	Breast density	DMs + ResNet18	27,684	Pretrained ResNet18	27,684	5741 + 1076	8738	Kappa = 0.67	NA	NA	77
Byra et al. [76]	2019	Classification of Breast US	USA	Breast US	INbreastes	582	AUC (VGG19 + FT + ML)	582	150	150	0.936	NA	NA	NA
Fujioka et al. [77]	2019	Breast mass segmentation	Japan	Breast US	DCNN	947	DCNN	480 benign, 467 cancerous	NA	NA	0.913	NA	NA	NA
E.P.V Le [78]	2019	classification	Uk	Breast imaging	iCAD’s Detection + ScreenPoint MedicalTranspara	640,000	CNN + DREAM Challenge	318,000	NA	NA	0.91 0.76	84.0	91 [79]	NA
Kayode et al. [80]	2019	SVM classifier	Nigeria	Breast	GLCM	322	Texture Feature + SVM	126 Normal, 60 benign, 48 malignant	NA	NA	NA	94.5	91.3	NA
Tomoyuki Fujioka, Kazunori Kubota et al. [77]	2019	Classification	Japan	Breast tumor	CNN	1536	CNN + Res152	897 malig, 639 benign	NA	NA	0.951	90.9	87.0	NA
Wei M [81]	2020	Classification	China	Breast tumor	Radial Range Spectrum + GLCM	1061	SVM classifier	589 malignant, 472 benign	NA	NA	0.93	87.0	87.6	87.3
Tomoyuki Fujioka, Kazunori Kubota et al. [77]	2020	Differentiation	Japan	Breast cancer	CNN	576.6	CNN	48 benign, 72 malignant	NA	NA	0.93, 0.73, 0.85	95.8, 58.3, 91.7	92.5 60.4 77.1	92.5 65.8 79.2

**Table 3:** Comparison of the latest related studies.

Reference	Country	Topic	Aim	Methods Used	Result	Limitation
Dutta et al., 2021 [82]	USA	The classification of Triple-Negative Breast Cancer (TNBC) using DL methodology on Patient Derived Tumor Xenograft (PDX) and Tumor Probability Boundary Sensitivity of Radiomic Pipeline	DL is used in the automated pathway for the detection and quantification of TNBC PDX tumors from nonclinical weighted (T1W) images and weighted images (T2WI) of M RI	Manual comparison of delineation with U-Net, dense U-Net, Res-Net, recurrent residual U-Net (R2UNet), and dense R2U-Net	The compared networks had a score ranging between 1% and 3%	Time intensive. Variations in the results of repeated measurements cannot be reproduced.
Zhang et al., 2022 [83]	UK	Diagnostic and Surgical Applications of AI that Can Be interpreted or explained	Overcome AI’s explainable or interpreted (XAI) black box identity	Literary search from databases between 2019 to 2021	XAI is heading in several interesting directions.	Black box identity of some of the AI models
Roy et al., 2020 [84]	USA	Radiomic characteristics and resolution clinical weighted (T1W) image and weighted image (T2WI) from MRI	Radiomics characteristics were used in a combined clinical trial.	The study uses TNBC patients	Sixteen volume-dependent characteristics of radiomics were identified	Models used are genetically engineered PDXs are also used.
Roy et al., 2022 [85]	USA	Predicting response to neoadjuvant in TNBC by using a co-clinical FDG-PET (RadSig) radiomic signature	Optimization and identification of radiometric characteristics and therapeutic response	The study uses TNBC patients as well as PDXs	NB-RadSig has the highest prediction and therapeutic response.	SVM-RadSig NB-RadSig superior to Standardized uptake values of mean, maximum, and peak

## Data Availability

The data come from literary studies that were searched and reviewed between 2012 and 2022.
